# *Wolbachia*-mediated parthenogenesis induction in the aphid hyperparasitoid *Alloxysta brevis* (Hymenoptera: Figitidae: Charipinae)

**DOI:** 10.1128/aem.01308-25

**Published:** 2025-11-07

**Authors:** Jonathan Dregni, Amelia R. I. Lindsey, Mar Ferrer-Suay, Sabrina L. Celis, George E. Heimpel

**Affiliations:** 1 Department of Entomology, University of Minnesota 5635, St. Paul, Minnesota, USA; 2 Departament de Zoologia, Universitat de València 16781, València, Spain; UMR Processus Infectieux en Milieu Insulaire Tropical, Ste Clotilde, France

**Keywords:** *Aphelinus certus*, *Aphis glycines*, endosymbiont, parasitoid, sex ratio, thelytoky

## Abstract

Thelytokous parthenogenesis (thelytoky), in which females can produce female offspring without mating, can be caused by parthenogenesis-inducing endosymbiotic bacteria in the genus *Wolbachia*. This interaction is well known in hymenopteran parasitoids, where unfertilized eggs typically develop as males via haplodiploidy in the absence of parthenogenesis-inducing bacteria. We report on a case of thelytoky in *Alloxysta brevis* (Thomson) (Hymenoptera: Figitidae), a globally widespread aphid hyperparasitoid. A previous study had shown that sex ratios of this species collected in Minnesota (USA) were extremely female-biased, and we found here that unmated females reared from field-collected hosts produced female offspring without exposure to males. This result demonstrated thelytoky, and we tested for the role of bacterial endosymbionts by comparing offspring production of unmated females fed the antibiotic rifampicin to offspring production of control females not fed antibiotics. Antibiotic-fed females produced almost exclusively male offspring, and control females produced mainly females. This result showed that antibiotic treatment facilitated male production by unmated *A. brevis* females, thus implicating bacterial symbiosis in the expression of thelytoky. We then used full-length 16S rRNA sequencing to determine the identity of the symbiont. These analyses identified a *Wolbachia* strain from supergroup B and excluded other bacteria known to mediate parthenogenesis induction, such as *Cardinium* and *Rickettsia*. While *Wolbachia* had been previously detected by molecular analysis in this species, these are the first experiments demonstrating *Wolbachia*-mediated parthenogenesis in the figitid subfamily Charipinae. To our knowledge, this also constitutes the first documented case of endosymbiont-mediated thelytoky in any hyperparasitoid species.

**IMPORTANCE:**

Parthenogenesis induction in insects can have important environmental and economic consequences. This is especially true if pests or their natural enemies are affected. The case of *Alloxysta brevis* is of particular interest, as this species is a hyperparasitoid of aphids, meaning that they attack and kill primary parasitoids of aphids. The populations of many species of pest aphids are controlled by primary parasitoid species, and hyperparasitoids thus have the potential to interfere with this mechanism of control. The role of hyperparasitoid parthenogenesis in the suppression of aphids by primary parasitoids remains unexplored. Thus, the results of this set of studies provide a starting point for determining whether parthenogenesis-inducing *Wolbachia* in hyperparasitoids should be expected to improve or hinder biological control of pest aphids by primary parasitoids. The focus on *A. brevis* as a model for these questions could be particularly instructive, since it is a species of worldwide distribution that is involved in numerous economically important aphid–parasitoid interactions.

## INTRODUCTION

**Fig 1:**
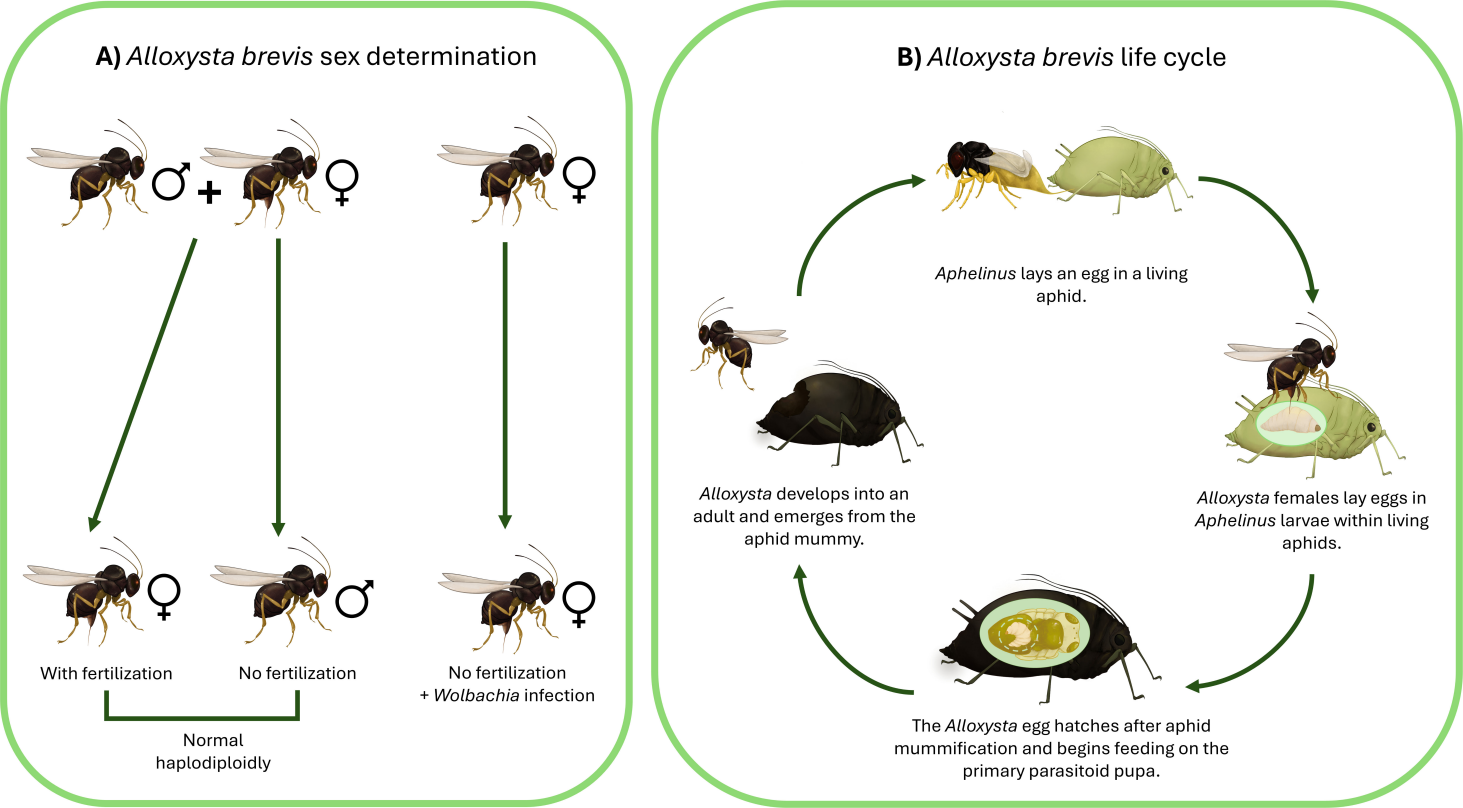
Sex determination and life cycle of *A. brevis*. (**A**) Patterns of normal haplodiploidy and endosymbiont-mediated parthenogenesis (**B**) Life cycle of *A. brevis* attacking an aphid that has previously been parasitized by the primary parasitoid *Aphelinus certus*. All figures drawn by Sabrina Celis.

Most species in the insect order Hymenoptera exhibit haplodiploid sex determination: unmated females produce only males from unfertilized eggs, while mated females can produce daughters by fertilizing some or all of their eggs. The production of daughters by unmated females is unusual, and within haplodiploid species, a distinction is made between arrhenotokous parthenogenesis (the typical haplodiploid system, where unmated females produce only sons) and thelytokous parthenogenesis (where unmated females produce daughters) (Fig. 1A). Thelytokous parthenogenesis (thelytoky) occurs in many species of parasitoid wasps and can arise either from genetic mechanisms or from the actions of parthenogenesis-inducing endosymbiotic bacteria (1–3). In the latter case, the presence of bacteria can alter meiosis or mitosis to cause the production of diploid embryos in the absence of fertilization, instead of haploid embryos that would develop as males under haplodiploidy sex determination (4–6). In parasitoid wasps, symbiont-induced parthenogenesis has been documented in the hymenopteran superfamilies Chalcidoidea, Cynipoidea, Ichneumonoidea, and Platygastroidea (1, 7). While most documented cases of symbiont-induced parthenogenesis can be attributed to bacteria in the genus *Wolbachia*, other bacteria such as *Cardinium* and *Rickettsia* have also evolved parthenogenesis-inducing mechanisms (2, 4).

Here, we determine whether the aphid hyperparasitoid *A. brevis* (Thomson, 1862) (Hymenoptera: Cynipoidea: Figitidae: Charipinae) exhibits symbiont-induced thelytoky, and if so, which microbe(s) are involved. Hyperparasitoids are parasitoids that attack other parasitoid species (8), and *A. brevis* is a broadly distributed species that uses a broad range of aphid parasitoids as hosts (9–12). We encountered *A. brevis* attacking soybean aphids, *Aphis glycines* Matsumura (Hemiptera: Aphididae) already parasitized by the parasitoid wasp *Aphelinus certus* Yasnosh (Hymenoptera: Aphelinidae) in soybean fields of Minnesota, USA (13). Of 264 adult *A. brevis* individuals reared, 263 were females and one was a male (13). Such an extremely biased sex ratio suggests thelytoky, and we used an experiment comparing the offspring of antibiotic-fed females with offspring of control females to evaluate the hypotheses that thelytoky was, in fact, operating in *A. brevis*, and if so, whether it could be attributed to endosymbiotic bacteria. Having found this to be the case, we used molecular analyses to identify the microbial taxon (or taxa) involved. The presence of *Wolbachia* DNA was previously reported from one *A. brevis* specimen from Norway (14). In this study, we confirm the presence of *Wolbachia* and show that a symbiont is mediating thelytoky in this species.

## MATERIALS AND METHODS

### Natural history of *Alloxysta* hyperparasitism

*Alloxysta* species are obligate aphid hyperparasitoids that oviposit into developing larvae of primary aphid parasitoids, including members of the genus *Aphelinus* (Hymenoptera: Aphelinidae) and of the subfamily Aphidiinae (Hymenoptera: Braconidae) (8; see Fig. 1B). A single egg is likely laid in most cases, but even in the case of multiple eggs being laid by a single female, or superparasitism (when different females oviposit into the same host), only a single adult *Alloxysta* emerges. *Alloxysta* eggs remain dormant until the primary parasitoid has pupated within a cocoon formed by the exoskeleton of the dead aphid (the ‘mummy’). The *Alloxysta* larva then consumes the pupa of the primary parasitoid internally and emerges from the aphid mummy (8).

### Review and analysis of *Alloxysta brevis* sex ratios

*A. brevis* is a globally distributed species, with samples collected in Africa, throughout Eurasia, as well as parts of the Neotropics and North America. We compiled collections of this species used for taxonomic studies and tabulated the adult sex ratios associated with each collection. We report the proportion of females per geographical location and sum over regions. For any data sample consisting of greater than 20 individuals, we used a two-tailed binomial test to determine whether the sex ratio was significantly biased toward females or males.

### Field sampling and rearing hyperparasitoids

*A. brevis* were reared from field-collected *Aphelinus certus* mummies, collected along a 600 km transect within the soybean-growing area of Minnesota, USA, during summer 2018 using methodologies described by Casiraghi et al. (13). The mummies were then held in a growth chamber at 24.5°C±1°C, at a 16:8 L:D photoperiod and observed daily for emergence. Hyperparasitoids emerging from *Aphelinus certus* mummies were identified to species by M.F.-S. and sexed by use of morphological characters using a key to the Charipinae (15). A colony was established by adding *A. brevis* females to plexiglass cages (30×38×36 cm) in which potted soybean plants (stages V1–V3) were seeded with soybean aphids and *Aphelinus certus* (16).

### Assessment of thelytoky

**Fig 2:**
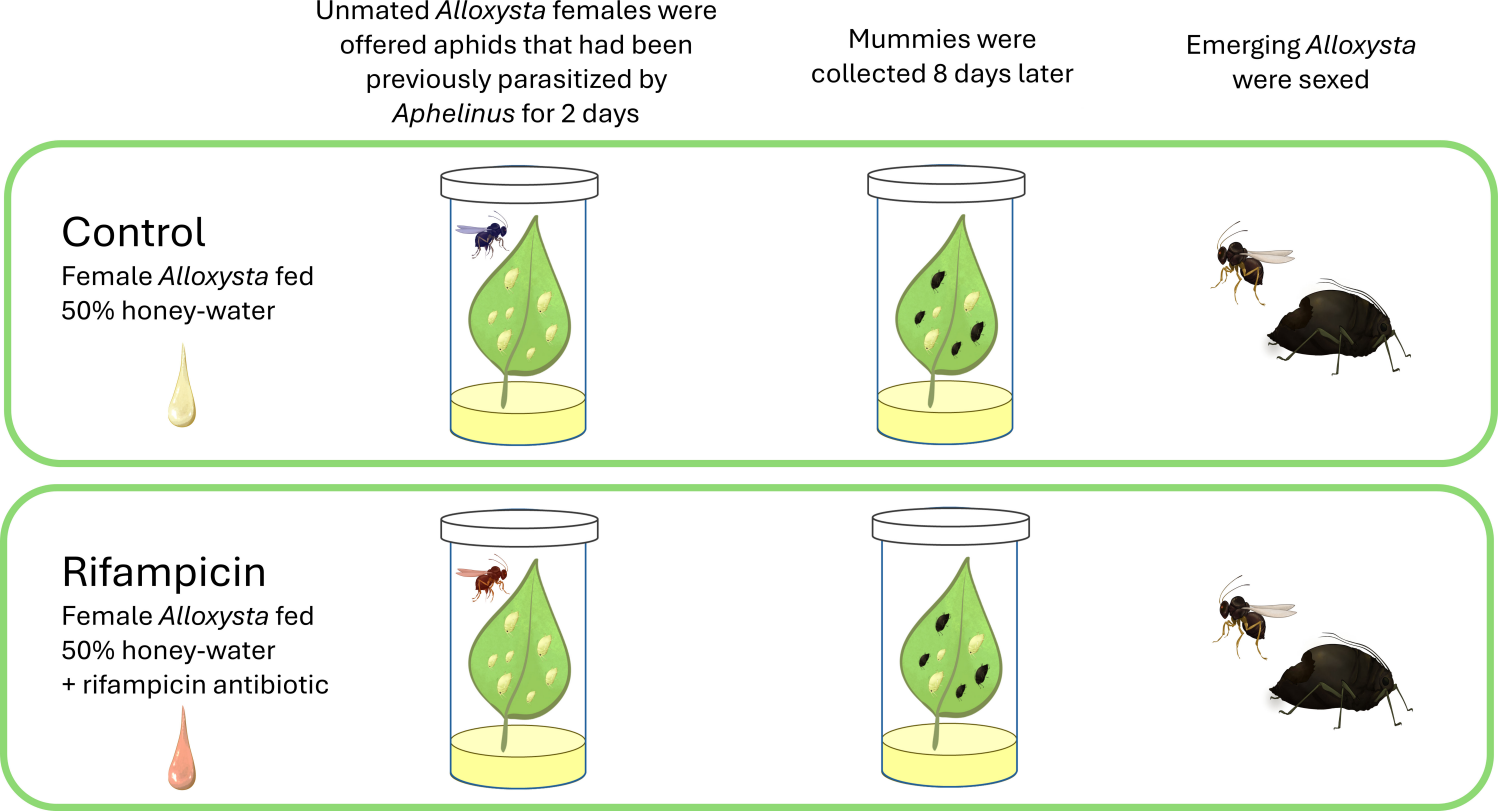
Experimental protocol. Schematic of protocol for the antibiotic-feeding experiment. All figures drawn by Sabrina Celis.

To conduct an initial assessment of parthenogenesis in *A. brevis*, a sample of 23 females that emerged from field-collected *Aphelinus certus* mummies were held individually upon emergence and exposed to soybean aphids parasitized by *Aphelinus certus* from a laboratory colony. We infested potted soybean plants with soybean aphids and added 8 *Aphelinus certus* adults. After 6–9 days, we harvested leaflets containing at least 2 *Aphelinus* mummies and 20 aphids, an unknown number of which contained *Aphelinus certus* larvae of various ages. These aphids were exposed to *A. brevis* females by placing the leaflet petiolule into a centimeter of damp sand in a 30 mL capped vial with a few droplets of 50% honey solution as a food source. We then added a single unmated, 3-day-old *A. brevis* female to each vial and moved her to vials containing fresh leaflets with hosts as described above every 2 days (Fig. 2). All aphid mummies were collected once they formed and placed singly into 0.6 mL microcentrifuge tubes and observed for parasitoid emergence. Since none of the *A. brevis* females used in this study had any exposure to males, the production of female offspring would be proof of thelytoky (2, 5).

### Antibiotic treatment

We compared the reproduction of *A. brevis* females fed the antibiotic rifampicin to control females (Fig. 2). Rifampicin was diluted in aqueous dimethyl sulfoxide at a concentration of 10 mg/mL and then mixed with honey and water to a final concentration of 1 mg/mL in 50% honey. Tiny (<0.5 mm diameter) droplets of control (no rifampicin) or rifampicin honey were placed in 0.6 mL microcentrifuge tubes containing *A. brevis* females that had emerged within the previous 24 h, and then held for 72 h at 26.2°C±0.5°C. After this period, each *A. brevis* female was placed in a soybean-leaf arena with up to 20 *Aphelinus certus*-parasitized soybean aphids as described above. No antibiotic treatment was applied after the initial 72 h feeding period, but all arenas were provided 50% control honey from this point onwards. *A. brevis* females were moved to fresh soybean leaflets containing parasitized soybean aphids as described above every 2 days for 12 days, resulting in six sequential assessments of reproduction for each experimental female. We evaluated 61 *A. brevis* females, alternately assigned either rifampicin honey (‘treated’) or untreated honey (‘control’), of which 19 treated and 16 control females reproduced during the course of the assay. Mummies that developed on the leaves were isolated as described above, and offspring species and sex were recorded upon emergence. We used two-tailed Fisher’s exact tests to determine whether there was a significant effect of the antibiotic treatment on male production, and whether there was a significant change in male production over the six time periods in the control treatment.

### Identification of bacterial endosymbiont

DNA was extracted from individual *A. brevis* specimens using HotSHOT (17) in a final total volume of 24 µL, as implemented previously for other microhymenopterans (18). *Wolbachia*-specific 16S PCRs were performed with WspecF and WspecR primers (19), and general bacterial full-length 16S PCRs leveraged 27F and 1,492R primers (20) (Table 1). All PCRs were prepared in a final volume of 20 µL with 1 µL of DNA template, Q5 Hot Start High-Fidelity 2× Master Mix (New England BioLabs), and 500 nM of each primer, alongside positive and negative controls. Negative controls included no-template PCR reactions, and a negative control for environmental contamination consisting of the extraction buffer mix alone into which the wasp-handling paintbrush was dipped. Thermal cycling was conducted on a Mastercycler nexus PCR cycler (Eppendorf) with an initial denaturation of 2 min at 98°C, 35 cycles of amplification (see Table 1), and final extension of 2 min at 72°C. PCR products were separated with electrophoresis on a 1% agarose gel and visualized under ultraviolet light after staining with GelRed (Biotium) diluted to 3× in water. Prior to sequencing, PCR products were cleaned using the Zymo DNA Clean & Concentrator – 5 Kit (Zymo Research), and quantification was performed with a Qubit 4 Fluorometer and the Qubit 1× dsDNA HS Assay Kit (Invitrogen).

**TABLE 1:** Information on primer pairs used to amplify *Wolbachia* sequences in *A. brevis*

Primer	Sequence (5′ → 3′)	Target	Cycling conditions	Citation
WspecF	CATACCTATTCGAAGGGATAG	*Wolbachia* 16S rRNA	15 s at 98°C, 15 s at 60°C, 15 s at 72°C	(18, 19)
WspecR	AGCTTCGAGTGAAACCAATTC			
27F	AGAGTTTGATCMTGGCTCAG	General Bacterial 16S rRNA	20 s at 98°C, 30 s at 58°C, 45 s at 72°C	(19, 20)
1492R	TACGGYTACCTTGTTACGACTT			

Full-length 16S PCR products derived from eight individual *A. brevis* individuals were sequenced via the ‘Premium PCR Sequencing’ service by Plasmidsaurus using Oxford Nanopore Technology, alongside extraction negative controls. Fastq files generated by Plasmidsaurus were then analyzed following our own custom pipeline. Specifically, reads were first taxonomically classified using the Oxford Nanopore Technologies EPI2ME ‘wf-16s’ v1.1.3 workflow employing Kraken2 v2.1.2 (21). Then, reads corresponding to taxa not present in the negative extraction control were extracted with custom bash scripts and assembled with the EPI2ME ‘wf-amplicon’ v1.0.4 workflow, which aligns and polishes reads to generate a consensus sequence using miniasm v0.3-r179 (22) and medaka v1.11.1. Consensus sequences and mapped reads (e.g., bam files) were manually inspected in JBrowse (23) to check for chimeric assemblies. The final *Wolbachia* 16S sequence from *A. brevis* was queried against the NCBI GenBank database with blastn and default parameters. 16S sequences from top GenBank matches, plus those from a range of other well-described *Wolbachia* strains (Table 2), were aligned with the secondary structure-aware SINA v1.2.12 aligner (24). Phylogenetic reconstruction was performed with IQ-TREE v1.6.11 (25), including model optimization (TPM3u + F + I selected), outgroup specification, and 1,000 rapid bootstraps.

**TABLE 2:** 16S DNA sequences used in phylogenetic reconstruction of *Wolbachia* sequences associated with various host species, indicating the *Wolbachia* supergroup (26, 27) and NCBI accession number

Strain	Host	Supergroup	Accession
*w*AlbA	*Aedes albopictus*	A	CP101657.1
*w*AlbB	*Aedes albopictus*	B	CP041923.1
*w*Au	*Drosophila simulans*	A	CP069055.1
wBm^*a*^	*Brugia malayi*	D	AJ010275.1
*w*Bpra	*Bryobia praetiosa*	B	EU499317.1
*w*Cpunc	*Cyclophora punctaria*	B	OZ034749.1
*w*DacB	*Dactylopius coccus*	B	LSYY01000126.1
*w*Di	*Diaphorina citri*	B	CP048820.1
*w*Dmed	*Dolichovespula media*	B	CAMXBR010000003.1
*w*Ha	*Drosophila simulans*	A	CP003884.1
*w*Lcla	*Leptopilina clavipes*	B	QJHA01000026.1
*w*Ma	*Drosophila simulans*	B	CP069054.1
*w*Mau	*Drosophila mauritiana*	A	CP034335.1
*w*Mel	*Drosophila melanogaster*	A	CP046925.1
*w*No	*Drosophila simulans*	B	CP003883.1
*w*Oo^*a*^	*Onchocerca ochengi*	C	AJ010276.1
*w*Pip	*Culex quinquefasciatus*	B	AM999887.1
*w*Suz	*Drosophila suzukii*	A	CAOU02000038.1
*w*Tei	*Drosophila simulans*	A	CP069052.1
*w*Tpre	*Trichogramma pretiosum*	B	LKEQ01000006.1
*w*Uni	*Muscidifurax uniraptor*	A	L02882.1
CIXPIL1	*Cixidia pilatoi*	B	OQ102143.1
MALBOS1	*Malenia bosnica*	B	OQ102152.1
PARIOC1	*Paracorethrura iocnemis*	B	OQ102158.1
PENROR1	*Pentastira rorida*	B	OQ102146.1

^*a*^ Indicates outgroups.

## RESULTS

### Global patterns of *Alloxysta brevis* sex ratios

The overall sex ratio of *A. brevis* adults collected across the globe was significantly female-biased (Table 3). This included cases of all-female samples of greater than 20 individuals in Africa, France, and Russia, and cases of significant, extreme female bias (>90%) in Japan, Germany, the Nearctic region as a whole, and the state of Minnesota within the United States, as well as more modest (but still significant) female-biased samples from the Balkans (Table 3).

**TABLE 3:** Numbers of female and male *A. brevis* collected globally, along with the proportion females from each sample^*a*^

Region/country	Female	Male	Prop. female	Reference
Africa (sum)	32	0	1.00^***^	(28, 29)
Morocco	30	0	1.00	(28, 29)
Zimbabwe	2	0	1.00	(28)
Asia (sum)	77	14	0.84^***^	
China	2	0	1.00	(30)
India	3	2	0.60	(30)
Iran	15	7	0.68	(29, 31)
Japan	56	5	0.92^***^	(29, 30)
Thailand	1	0	1.00	(30)
Europe (sum)	542	110	0.83^***^	
Balkan Peninsula	153	85	0.64^***^	(32, 33)
Croatia	1	0	1.00	(29)
Czech Republic	11	1	0.92	(29)
France	23	0	1.00^***^	(29, 34)
Germany	269	10	0.96^***^	(29); M. Ferrer-Suay, unpublished data
Greece	3	0	1.00	(29)
Italy	13	7	0.65	(11)
Poland	11	0	1.00	(35)
Russia	23	0	1.00^***^	(29)
Serbia	2	0	1.00	(29)
Spain	3	1	0.75	(29)
Slovakia	1	0	1.00	(29)
Slovenia	9	4	0.69	(29)
Sweden	4	0	1.00	(29)
Switzerland	16	2	0.89	(29)
Neotropical (sum)	8	1	0.89	
Ecuador	1	0	1.00	(28)
Guatemala	1	0	1.00	(28)
Mexico	6	1	0.86	(36)
Nearctic (sum)	296	4	0.99^***^	
USA: California	16	0	1.00	(10)
USA: Colorado	6	1	0.85	(10)
USA: Iowa	2	2	0.50	(10)
USA: Georgia	3	0	1.00	(10)
USA: Maryland	2	0	1.00	(10)
USA: Minnesota	263	1	0.99^***^	(13)
USA: Mississippi	1	0	1.00	(10)
USA: Montana	1	0	1.00	(10)
USA: Utah	1	0	1.00	(10)
USA: Washington	1	0	1.00	(10)
Total	951	129	0.88^***^	

^*a*^ Significant differences from equal sex ratios are indicated for samples comprising at least 20 individuals using two-tailed binomial tests: ^*^P < 0.05, ^**^P < 0.01, ^***^P < 0.001 (10, 11, 13, 29–35).

### *Alloxysta brevis* reproduces via symbiont-mediated thelytokous parthenogenesis

**Fig 3:**
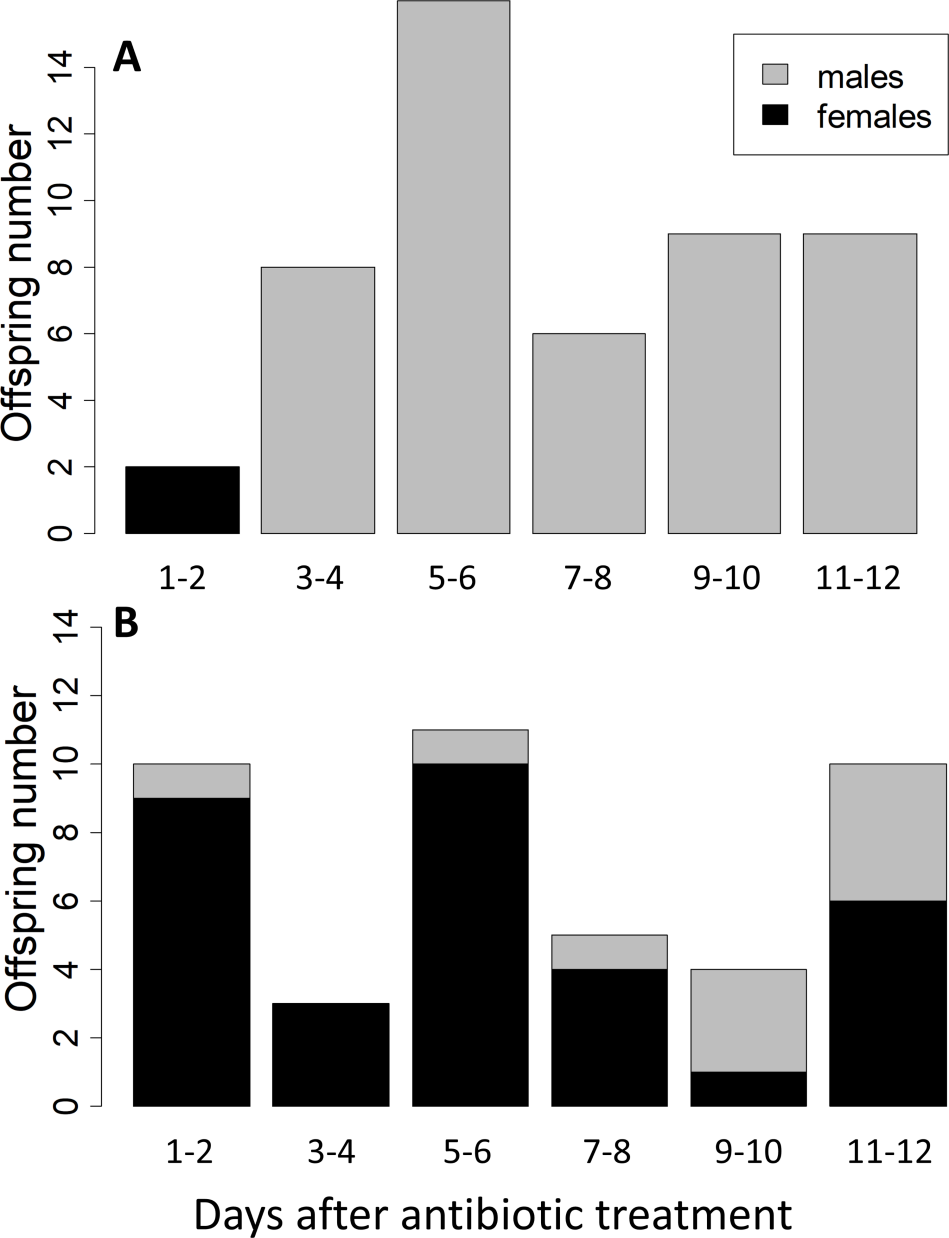
Antibiotic treatment interferes with thelytoky in *A. brevis*. (**A**) Numbers of male and female offspring produced by 19 females treated with the antibiotic rifampicin over 12 days after treatment. (**B**) Numbers of male and female offspring produced by 16 control females not treated.

Of 23 field-collected, unmated, *A. brevis* females reared from *Aphelinus certus* mummies, 16 produced 61 offspring, all of which were daughters (3.8 ± 0.9 [SEM] daughters per female). This result indicated that these parasitoids reproduce via thelytoky, so next, we used antibiotic treatment to determine if this reproductive mode was mediated by a bacterium. In total, the antibiotic-treated females produced 2 females and 47 male offspring, while the control females produced 33 females and 10 males (Fig. 3). This is a highly significant increase in male production by treated females (two-tailed Fisher’s exact test; *P* < 0.0001, odds ratio = 71.8 with 95% confidence interval of 14.8–705.3). Furthermore, the daughters produced by antibiotic-treated females were only produced during the first 48 h oviposition period after antibiotic treatment, while the control females produced daughters throughout the 12-day experimental period (Fig. 3). There was a marginally significant trend for a decreasing female bias over the six time periods in the control group (Two-tailed Fisher’s exact test: *P* = 0.079; Fig. 3B), a pattern that has been noted for other parasitoids that reproduce via symbiont-mediated parthenogenesis (37).

### *Alloxysta brevis* is associated with *Wolbachia* from Supergroup B

**Fig 4:**
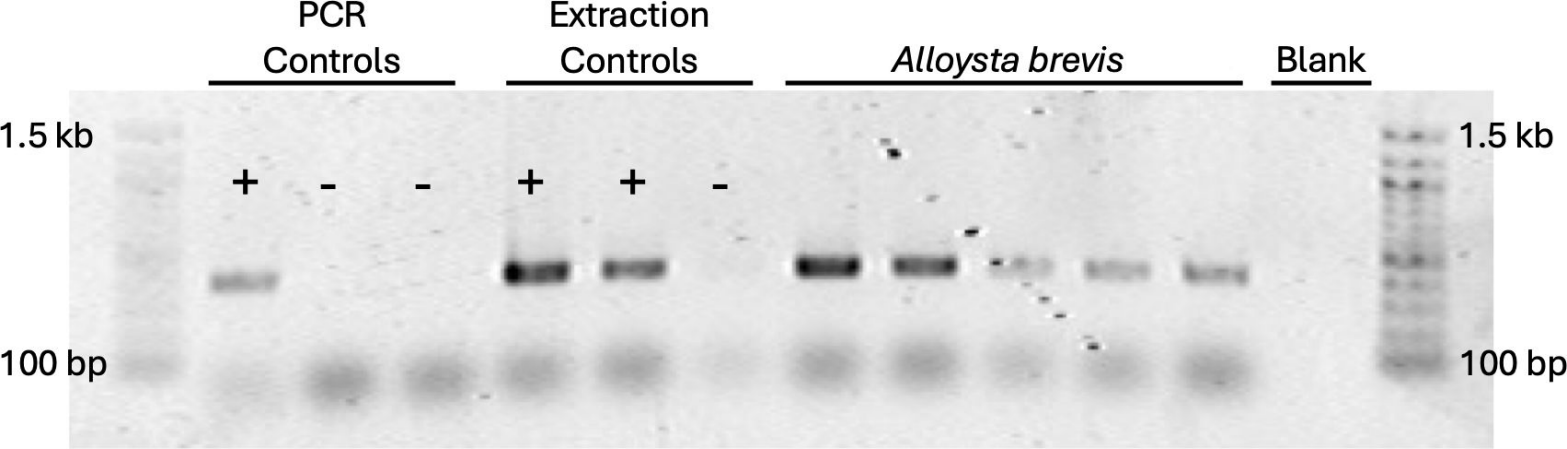
Diagnostic PCR for *Wolbachia. Wolbachia*-specific 16S “Wspec” primers (18) were used to test for the presence of *Wolbachia* DNA in individual *A. brevis* wasps. In total, eight individual *A. brevis* were screened, all positive for *Wolbachia* DNA. The results of five individuals are shown here. PCR controls included (+) a previously tested DNA extraction from *Wolbachia*-infected *Trichogramma pretiosum* (4) and no template (-) reactions. Extraction controls included PCR amplifications of fresh extractions from wasps of the same colony of *Trichogramma pretiosum* (+), and a negative extraction control (-), which included extraction buffers into which the wasp-handling paintbrush was dipped.

**Fig 5:**
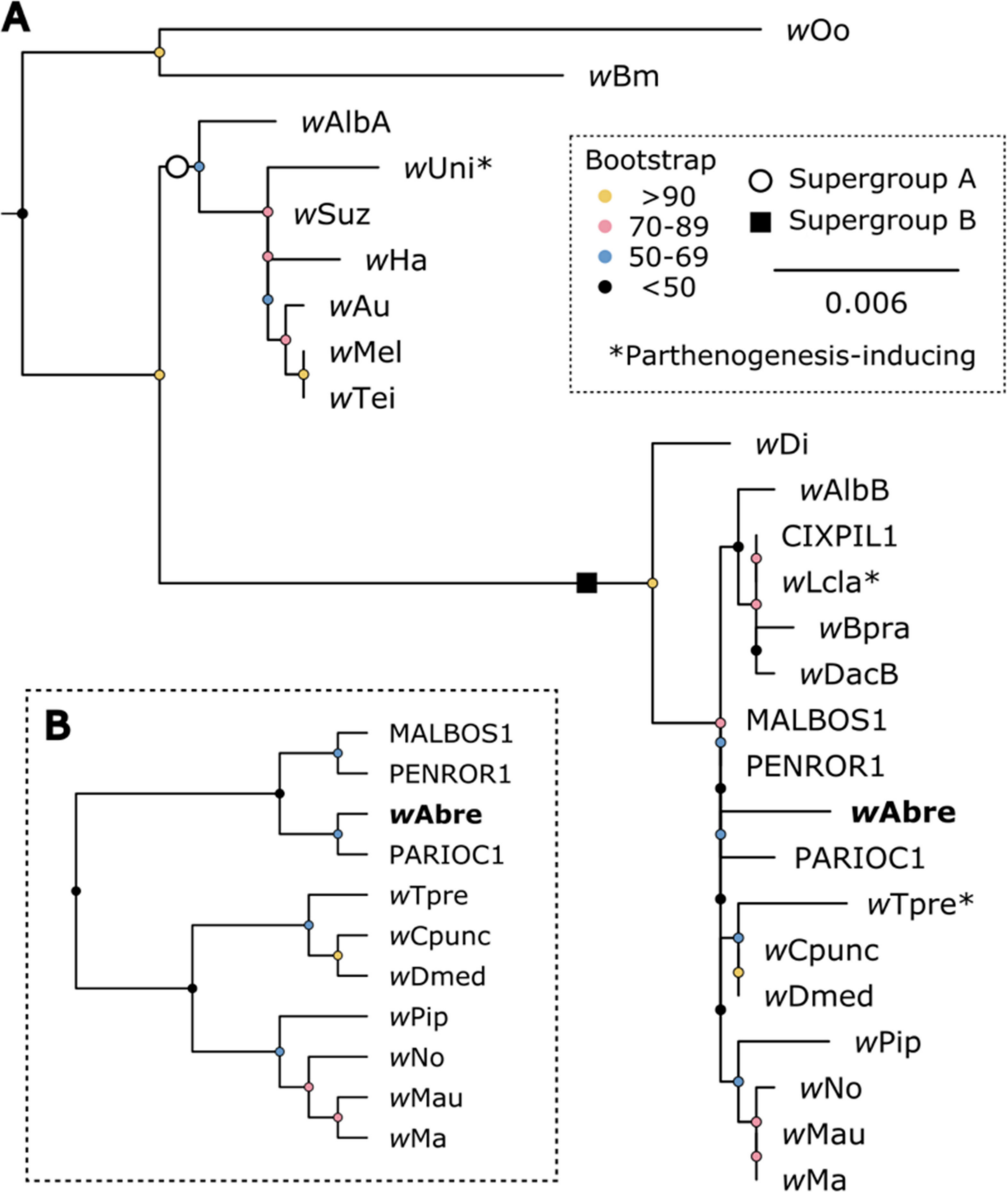
*Wolbachia* strain *w*Abre nests within Supergroup B. (**A**) Phylogenetic reconstruction of *Wolbachia* based on full-length 16S rRNA. (**B**) Monophyletic group within supergroup B containing *w*Abre, displayed as a cladogram to facilitate better interpretation of relationships given the especially short branch lengths in (**A**).

*Wolbachia*-specific PCR assays indicated that all *A. brevis* individuals tested (*n* = 8) were positive for *Wolbachia* DNA (Fig. 4). We then used amplicon sequencing of full-length 16S to (1) check for the presence of other symbionts, and (2) determine if there were multiple *Wolbachia* strains present. After removal of environmental contaminants, *Wolbachia* was the only insect-associated microbe in the sequencing data, and assembly of all reads that were assigned to order Rickettsiales resulted in a single high-confidence contig. Given that multiple Rickettsiales (e.g., *Wolbachia* and *Rickettsia*, or perhaps two *Wolbachia* strains) could have been assembled into a single contig, we manually inspected read alignments. Any variants (as compared to the consensus sequence) were (1) present at low and inconsistent frequencies, and (2) did not consistently co-occur in a single read (i.e., there was no support for a second low-frequency haplotype), indicating that there was a single *Wolbachia* strain across all the individuals we screened. Phylogenetic analyses indicated that this *Wolbachia* strain from *A. brevis*, hereinafter ‘*w*Abre’, is closely related to other *Wolbachia* from ‘Supergroup B’ (Fig. 5).

## DISCUSSION

*A. brevis* females, collected as immatures in soybean fields in Minnesota, USA, and then isolated in the laboratory with no opportunity to mate, produced only daughters. This result confirms the hypothesis of thelytoky in this population of *A. brevis*, which had been previously inferred based on an extremely female-biased sex ratio (13). Thelytoky in haplodiploid species such as the Hymenoptera can be caused by genetic mechanisms or by parthenogenesis-inducing bacterial endosymbionts (1, 2), and we showed that treatment with antibiotics led to the production of male offspring. This demonstrates that bacterial associates are involved in causing thelytoky in this parasitoid species. Further, our molecular analyses identified that the endosymbiotic bacterium *Wolbachia* was associated with *A. brevis* females. One caveat is that PCR-based assays can result in false positives when *Wolbachia* DNA is integrated into the host genome (38, 39). However, given the results of the antibiotic treatments, and since 16S amplicon sequencing did not recover any other bacterial taxa, we conclude that *Wolbachia* likely mediates parthenogenesis induction in the aphid hyperparasitoid *A. brevis*. The finding of *Wolbachia*-mediated parthenogenesis induction in *A. brevis* is not particularly surprising, as at least three members of the family Figitidae exhibit this association (two *Leptopilina* species*,* and *Gronotoma micromorpha* [Perkins] [2, 38, 40, 41]).

Thelytoky refers specifically to the ability of unmated females to produce daughters and does not exclude the production of males under some conditions. While all of our field-collected *A. brevis* produced only daughters when held at 24.5°C, the control females in our antibiotic-treatment assay produced appreciable numbers of sons (10 males out of 43 offspring produced). For this second experiment, females in both treatments were held at 26.2°C for 3 days prior to exposure to hosts to improve the effectiveness of the antibiotic rifampicin for the treated parasitoids (see 38, 41, 42). Elevated temperatures are known to ‘cure’ insects of *Wolbachia* endosymbionts, with even temperatures between 25°C and 27°C leading to reductions in *Wolbachia* titer (43). We thus hypothesize that elevated temperature was at least partially responsible for male production in the control treatment of this experiment. Another observation worth noting is that female offspring were produced by treated females over the first 2 days of the assay. This is not unexpected as the first eggs laid by treated females often do not show signs of endosymbiont elimination in parasitoids (38, 43, 44). This is presumably due to the fact that female parasitoids often eclose as adults with one or more chorionated eggs (45) that would likely be inaccessible to antibiotics ingested by the female. Furthermore, even if the microbe were eliminated in these mature eggs, the treatment would not eliminate already secreted bacterial effector proteins that drive the developmental changes.

*A. brevis* exhibits female-biased sex ratios in collections from throughout the world, some of which consist exclusively of females. While our experiments show that *Wolbachia*-mediated thelytoky likely contributes to this global trend, other causes, particularly adaptive patterns of sex allocation by mated females (46), cannot be ruled out. Previous laboratory work with *A. brevis* using individuals collected in Germany used both females and males, with no mention of thelytoky or female-biased sex ratios (47, 48). This suggests that arrhenotokous populations of *A. brevis* occur without the intervention of antibiotic treatment. Thus, while we have demonstrated thelytoky in a population of *A. brevis* collected in Minnesota, USA, the broader literature on this species suggests a scenario in which both thelytokous and arrhenotokous populations exist. This is not unexpected, as it has been seen in other *Wolbachia*-parasitoid interactions such as *Trichogramma* species in California (49, 50). In the Figitidae, *Leptopilina clavipes* (Hartig) includes thelytokous populations in the Netherlands and an arrhenotokous population in Spain (41). In this case, Dutch parasitoids tested positive for *Wolbachia* while Spanish parasitoids did not. We hypothesize a similar scenario for *A. brevis*, with the presence of *Wolbachia* (and therefore thelytokous populations) facilitated by conditions that are permissive of *Wolbachia*, or relatively recent local acquisition of *Wolbachia* in some populations.

Since *A. brevis* is a hyperparasitoid of aphids, its association with parthenogenesis-inducing *Wolbachia* may affect its capacity to disrupt biological control of pest aphid species. To our knowledge, this represents the first documented case of endosymbiont-mediated thelytoky in a hyperparasitoid species. Consequently, the potential implications of this phenomenon for the biological control of pest species have received little attention. Female-biased sex ratios *per se* are expected to increase the effectiveness of parasitoids in suppressing host populations (51–53). For hyperparasitoids, this would suggest a trend for increased disruption of biological control for hyperparasitoids as the female bias increases. However, harboring parthenogenesis-including *Wolbachia* can also result in lower total reproductive output (54, 55), and the ability of *Wolbachia* to alter offspring sex ratio itself can vary with reproductive rates (2). It is thus not clear how the association with parthenogenesis-inducing *Wolbachia* that we have uncovered for *A. brevis* should be expected to influence aphid biological control. Further studies comparing populations of this parasitoid that do or do not harbor *Wolbachia* under varying conditions of host availability could shed light on this question. Given the widespread geographic distribution of this parasitoid species, and its broad host range (10, 12), such studies could be of broad relevance to aphid biological control.

## Data Availability

The full-length *w*Abre 16S sequence has been deposited in GenBank under accession number PV568413.1.
